# Simvastatin is a potential candidate drug in ovarian clear cell carcinomas

**DOI:** 10.18632/oncotarget.27747

**Published:** 2020-10-06

**Authors:** Nicolai Skovbjerg Arildsen, Ingrid Hedenfalk

**Affiliations:** ^1^Division of Oncology, Department of Clinical Sciences, Lund and Lund University Cancer Center, Lund University, Lund, Sweden; ^2^Current Address: Leo Foundation Skin Immunology Research Center, Department of Immunology and Microbiology, Faculty of Health and Medical Sciences, University of Copenhagen, Copenhagen, Denmark

**Keywords:** ovarian clear cell cancer, simvastatin, CID-1067700, Rho GTPase, actin

## Abstract

Ovarian clear cell carcinomas (OCCC) constitute a rare subtype of epithelial ovarian cancer, lacking efficient treatment options. Based on previous studies, we assessed the anti-proliferative effect of simvastatin, a Rho GTPase interfering drug, in three OCCC cell lines: JHOC-5, OVMANA and TOV-21G, and one high-grade serous ovarian cancer (HGSOC) cell line, Caov3. We used the Rho GTPase interfering drug CID-1067700 as a control. All OCCC cell lines were more sensitive to single-agent simvastatin than the HGSOC cells, while all cell lines were less sensitive to CID-1067700 than to simvastatin. Combinations of carboplatin and simvastatin were generally antagonistic. Most treatments inhibited migration, while only simvastatin and CID-1067700 also disrupted actin organization in the OCCC cell lines. All treatments induced a G1 arrest in JHOC-5 and TOV-21G cells. Treatments with simvastatin consistently reduced c-Myc protein expression in all OCCC cell lines and displayed evidence of causing both caspase-mediated apoptotic cell death and autophagic response in a cell line dependent manner. Differences between cell lines in response to the treatments were observed and such differences, including e. g. prior treatment, should be investigated further. Conclusively, simvastatin efficiently controlled OCCC proliferation and migration, thus showing potential as a candidate drug for the treatment of OCCC.

## INTRODUCTION

Ovarian clear cell carcinoma (OCCC) is a subtype of epithelial ovarian cancer (EOC) accounting for 5–10% of cases diagnosed in Europe and America, while the incidence in Asia is reported to be higher (10–20%) [1, 2]. OCCC presents with a distinct morphology with large clear cells containing glycogen [3, 4] and is considered chemo-resistant [5]. Co-occurrence of *ARID1A* and *PIK3CA* mutations is common, leading to PI3K-AKT-mTOR pathway activation [6]. Loss-of-function mutations in *KRAS* and *PTEN* are also frequent [7]. OCCC often presents in early stages (I-II), and upfront radical surgery is the primary treatment modality. However, following relapse the overall 5-year survival is shorter than for patients with the predominant EOC subtype, high-grade serous ovarian cancer (HGSOC) [8, 9].

We recently reported Rho (Ras homologous) GTPases and their associated pathways to be differentially expressed between OCCC compared to the other major EOC subtypes (HGSOC, endometrioid and mucinous ovarian cancers) [10]. Rho GTPases constitute one of five sub-families of the Ras small GTPase superfamily (Rho, Ras, Rab, Ran, Arf). Together they couple extracellular signals to intracellular signaling networks, thereby exerting their roles as both mediators and regulators within the cell [11]. Rho GTPases have been studied as targets for cancer treatment in various settings due to their role in regulating key cellular functions including the maintenance of cytoskeletal integrity, cell migration and proliferation [12–14], but also in metastasis and progressive disease in many cancer types [15, 16]. Furthermore, Rho GTPases have been implicated in carboplatin resistance in EOC [17]. However, targeting Rho GTPases directly is challenging due to their high binding affinity for GTP/GDP, and indirect strategies such as targeting the localization of Rho GTPases to the cell membrane are promising alternatives [18].

Statins inhibit the conversion of HMG-CoA into mevalonic acid, and thus inhibit the synthesis of the isoprenoid intermediates farnesylpyrophosphate (FPP) and geranylgeranyl pyrophosphate (GGPP), the latter of which is required by Rho GTPases for localization to the membrane [19]. Although debated, some evidence for increased survival in EOC patients after statin treatment has been shown, while the effect upon EOC risk is unclear [20–22]. Statins have however shown potential as an anticancer drug in ovarian cancer with most interest in HGSOC [23–26], while fewer reports have investigated statins in OCCC [20, 27]. CID-1067700 is a pan-GTPase inhibitor that inhibits binding of GTP/GDP and downstream binding of Rho GTPases to their targets [28] and is used as a comparator for Rho GTPase interference as a druggable target in OCCC. Based on the deregulated expression of both Rho GTPases and cytoskeletal pathways in primary human OCCC tumors in our previous work [10], we investigated the potential of simvastatin, a lipophilic statin, as a targeted treatment in OCCC cell lines with CID-1067700 as a comparator in the present study.

## RESULTS

### OCCC cell line characteristics

The characteristics of the OCCC cell lines used in this study, JHOC-5 [29], OVMANA [30] and TOV-21G [31] are summarized in Table 1.

**Table 1:** Cell line characteristics

	JHOC-5	OVMANA	TOV-21G
Origin and treatments			
Ethnicity	Japan	Japan	Canada
Stage	IIC	IV	III
Prior treatment	Yes (Cisplatin)	Yes (Cisplatin)	No
Tissue	Recurrent tumor (Pelvic)	Primary tumor	Primary tumor
Mutations and aberrations			
*ARID1A*	No	Yes	Yes
*PIK3CA*	No	Yes	Yes
*KRAS*	No	Yes	Yes
*PTEN*	No	Yes	Yes
Copy number aberrations [32]	Yes (*PTEN* loss)	Yes	No
Number of mutations reported [33]	308	519	1,708
Diagnostic markers			
HNF1-β	Positive	Positive	Positive
Napsin A	Negative	Positive	Negative

JHOC-5 cells are of Japanese origin, generated from a patient with a stage IIC recurrent pelvic tumor who had received prior chemotherapy treatment (cisplatin). JHOC-5 cells display copy number aberrations throughout the genome, affecting OCCC genes such as *PTEN* (loss) [32]. However, no mutations in genes commonly mutated in OCCC such as *ARID1A* or *PIK3CA* are reported [33]. JHOC-5 cells were found to be positive for HNF1-β, one of two clinical diagnostic markers for OCCC.

OVMANA cells, also of Japanese origin, were generated from a patient with a stage IV primary tumor who had received prior treatment (cisplatin). OVMANA cells also display copy number aberrations throughout the genome, in addition to harboring mutations in OCCC genes: *ARID1A*, *PIK3CA*, *KRAS* and *PTEN* [32, 33]. OVMANA cells were positive for both HNF1-β and Napsin A.

TOV-21G cells are derived from a treatment naïve patient from Canada with a stage III primary tumor. TOV-21G cells display no copy number aberrations but display a higher number of mutations throughout the genome (SNPs, insertions and deletions) compared to JHOC-5 and OVMANA. TOV-21G harbors mutations in *ARID1A*, *PIK3CA*, *KRAS* and *PTEN* [32, 33]. TOV-21G cells were found to be positive for HNF1-β.

Taken together, these three OCCC cell lines represent the heterogeneity observed in OCCC tumors in patients.

### Cytotoxic sensitivity to carboplatin, simvastatin and CID-1067700

**Figure 1:**
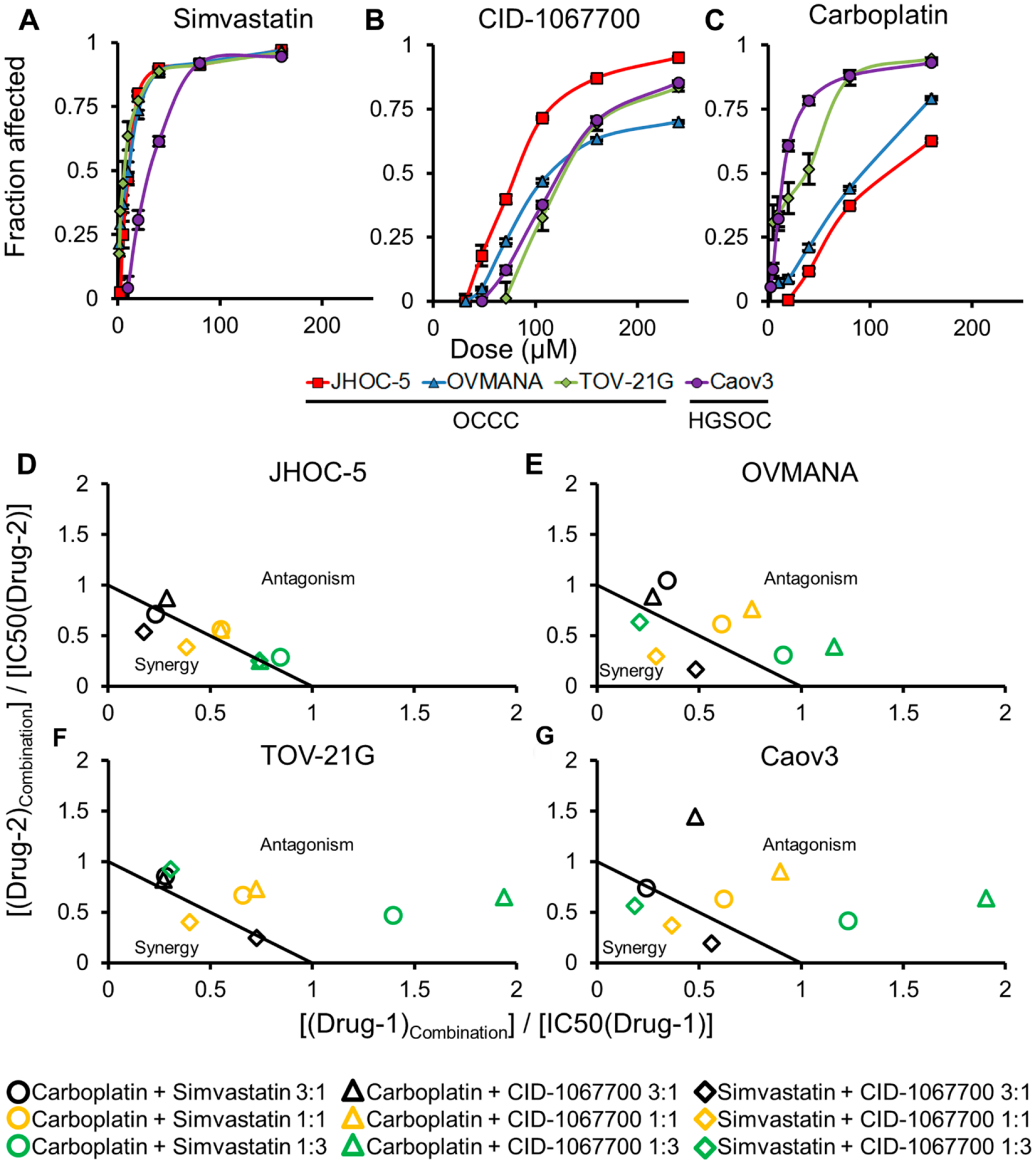
Concentration response curves (top panels) and normalized isobolograms (bottom panels) for treatments in cell lines. Top panels (**A**–**C**) are (A) Simvastatin; (B) CID-1067700; (C) Carboplatin; in JHOC-5 (red square), OVMANA (blue triangle), TOV-21G (green rhombus) and Caov3 (purple circle) cell lines. Bottom panels (**D**–**G**) are (D) JHOC-5; (E) OVMANA; (F) TOV-21G; (G) Caov3; isobolograms for carboplatin+simvastatin (circle), carboplatin+CID-1067700 (triangle) and simvastatin+CID-1067700 (rhombus) for ratios of 3:1 (black), 1:1 (orange) and 1:3 (green). Results are from at least three independent experiments in triplicate (*n* > 9). Error bars are standard error of the mean (SEM).

Single regimen drug responses after 72 hours of treatment with either carboplatin, simvastatin (concentration ranges 0–160 μM) or CID-1067700 (concentration ranges 0–240 μM) were evaluated in the three OCCC cell lines JHOC-5, OVMANA, TOV-21G and in the carboplatin sensitive HGSOC cell line Caov3 [34]. The sensitivities for single agent treatments with simvastatin, CID-1067700 and carboplatin are listed in Table 2 and concentration response curves are plotted in Figure 1A–1C).

**Table 2:** IC50 concentrations (μM) calculated and compared between cell lines using the drc-package, using a Bonferroni corrected significance threshold of 0.003 for 18 comparisons

IC50 (μM)			
	Simvastatin	CID-1067700	Carboplatin
JHOC-5	10.0 ± 0.8	81.4 ± 3.4	114.8 ± 9.2
OVMANA	7.2 ± 0.8	129.3 ± 7.3	84.5 ± 5.0
TOV-21G	5.6 ± 0.6	133.6 ± 5.0	17.8 ± 2.2
Caov3	31.2 ± 3.4	126.0 ± 7.6	16.6 ± 1.9
	**Comparisons of IC50 concentrations**		
		***p*-value **	
	**Simvastatin**	**CID-1067700**	**Carboplatin**
JHOC-5/OVMANA	0.0066	< 0.0001	0.0006
JHOC-5/TOV-21G	< 0.0001	< 0.0001	< 0.0001
JHOC-5/Caov3	< 0.0001	< 0.0001	< 0.0001
OVMANA/TOV-21G	0.0397	0.2842	< 0.0001
OVMANA/Caov3	< 0.0001	0.4229	< 0.0001
TOV-21G/Caov3	< 0.0001	0.0123	0.4088

The OCCC cell lines were more sensitive to simvastatin compared to the HGSOC Caov3 cells (Figure 1A, Table 2). JHOC-5 cells were significantly more sensitive towards CID-1067700 compared to the other three cell lines (Figure 1B, Table 2). TOV-21G and Caov3 cells were equally sensitive to carboplatin, while both JHOC-5 and OVMANA cells were less sensitive to carboplatin (Figure 1C, Table 2).

Overall, OCCC cell lines recapitulate the lower response to conventional carboplatin therapy compared to HGSOC cells observed in the clinic, while displaying significant sensitivity to treatment with simvastatin.

### Combination treatments with carboplatin, simvastatin and CID-1067700

Combinations between two drugs were assessed using the constant-ratio setup as suggested by Chou (2006) [35]. The results of the combination treatments are shown in Table 3 and Figure 1D–1G).

**Table 3:** IC50 concentrations and combination index values for combination treatments

JHOC5	Ratio	IC50 (μM)	Individual drug concentration (μM)			CI-Index
			Carboplatin	Simvastatin	CID-1067700	
Carboplatin + Simvastatin	3:1	100.1 ± 8.9	97.28	2.82		1.13 ± 0.1
	1:1	69.4 ± 5.1	63.84	5.56		1.11 ± 0.08
	1:3^a^	34.1 ± 2.7	27.04	7.06		0.94 ± 0.07
Carboplatin + CID-1067700	3:1^a^	105.6 ± 8.7	85.41		20.19	0.99 ± 0.08
	1:1	108.7 ± 7.5	63.60		45.10	1.11 ± 0.08
	1:3	103.6 ± 7.4	33.13		70.47	1.15 ± 0.08
Simvastatin + CID-1067700	3:1^a^	27.6 ± 1.2		7.43	20.17	0.99 ± 0.04
	1:1	35.1 ± 1.4		3.84	31.26	**0.77 ± 0.03**
	1:3	45.1 ± 2.2		1.77	43.33	**0.71 ± 0.03**
**OVMANA**						
Carboplatin + Simvastatin	3:1	79.4 ± 3.8	77.21	2.19		1.22 ± 0.06
	1:1	56.1 ± 3.2	51.70	4.40		1.22 ± 0.07
	1:3^a^	36.8 ± 2.3	29.31	7.49		1.39 ± 0.09
Carboplatin + CID-1067700	3:1	148.5 ± 8.9	98.34		50.16	1.55 ± 0.09
	1:1	162.4 ± 9.5	64.19		98.21	1.52 ± 0.09
	1:3^a^	137.3 ± 9.8	23.09		114.21	1.16 ± 0.08
Simvastatin + CID-1067700	3:1^a^	31.8 ± 2.5		4.55	27.25	**0.84 ± 0.07**
	1:1	39.6 ± 2.9		2.09	37.51	**0.58 ± 0.04**
	1:3	63.7 ± 6.6		1.16	62.54	**0.64 ± 0.07**
**TOV21G**						
Carboplatin + Simvastatin	3:1	27.5 ± 3.0	24.89	2.61		1.86 ± 0.02
	1:1	15.5 ± 1.4	11.79	3.71		1.32 ± 0.12
	1:3^a^	9.8 ± 0.8	5.04	4.76		1.13 ± 0.05
Carboplatin + CID-1067700	3:1	120.9 ± 32.0	34.52		86.38	2.59 ± 0.68
	1:1	109.9 ± 12.4	12.92		96.98	1.45 ± 0.16
	1:3^a^	114.2 ± 11.2	4.86		109.34	1.09 ± 0.11
Simvastatin + CID-1067700	3:1^a^	46.3 ± 3.0		5.20	41.10	1.23 ± 0.08
	1:1	55.7 ± 3.2		2.20	53.50	**0.80 ± 0.05**
	1:3	98.7 ± 6.1		1.40	97.30	0.97 ± 0.60
**CAOV3**						
Carboplatin + Simvastatin	3:1	33.3 ± 2.9	20.47	12.83		1.64 ± 0.14
	1:1	29.8 ± 2.5	10.35	19.45		1.25 ± 0.10
	1:3	27.0 ± 2.0	4.07	22.93		0.98 ± 0.07
Carboplatin + CID-1067700	3:1	111.8 ± 28.3	31.67		80.13	2.54 ± 0.64
	1:1	128.2 ± 15.8	14.92		113.28	1.80 ± 0.22
	1:3	190.0 ± 15.7	7.99		182.01	1.93 ± 0.16
Simvastatin + CID-1067700	3:1	41.1 ± 2.4		17.52	23.58	**0.75 ± 0.04**
	1:1	57.9 ± 2.7		11.49	46.41	**0.74 ± 0.03**
	1:3	76.7 ± 3.3		5.85	70.85	**0.75 ± 0.03**

^a^Combinations chosen for further study. Synergistic CI values (CI < 0.9) are in bold.

JHOC-5 and OVMANA cells showed similar patterns of sensitivity towards the combination treatments. Increasing the ratio of simvastatin also increased the sensitivity in both cell lines. Combinations of carboplatin and either simvastatin or CID-1067700 did not cause synergy in either cell line (Figure 1D and 1E (circle and triangle)), but combinations of carboplatin and simvastatin were more effective than carboplatin and CID-1067700 (Table 3). A 3:1 combination of simvastatin and CID-1067700 was the most effective of all combinations in both cell lines, while also showing synergy for most ratios (Table 3, Figure 1D and 1E (rhombus)).

TOV-21G and Caov3 cells showed similar patterns of sensitivity towards combination treatments. However, in contrast to JHOC-5 and OVMANA cells, combinations of carboplatin and simvastatin caused the highest effect on proliferation in these cell lines, but were found to be antagonistic regardless of ratio for all but one combination (Figure 1F and 1G (circle), Table 3). Combinations of carboplatin and CID-1067700 were antagonistic (Figure 1F and 1G (triangle), Table 3) for all combinations, while also having the least effect on the cells. Combinations of simvastatin and CID-1067700 were generally synergistic (Figure 1F and 1G (rhombus), Table 3). For both simvastatin combinations, an increase in the ratio of simvastatin led to an increased treatment effect (Table 3).

Taken together, responses to combination treatments varied across cell lines, but combinations of simvastatin and CID-1067700 were in general synergistic in OCCC cell lines.

### Evaluation of cytoskeletal integrity and cellular migration

Since Rho GTPases are known to regulate the cytoskeleton, we investigated the effects of the treatments on cytoskeletal integrity in the OCCC cell lines. In the following paragraphs, the plural word “treatments” in connection with an agent refers to the use of that agent alone and/or in any combinations where it occurs, unless otherwise noted. Conversely, “treatment” refers to a (single) specific agent or combination, unless otherwise noted.

**Figure 2:**
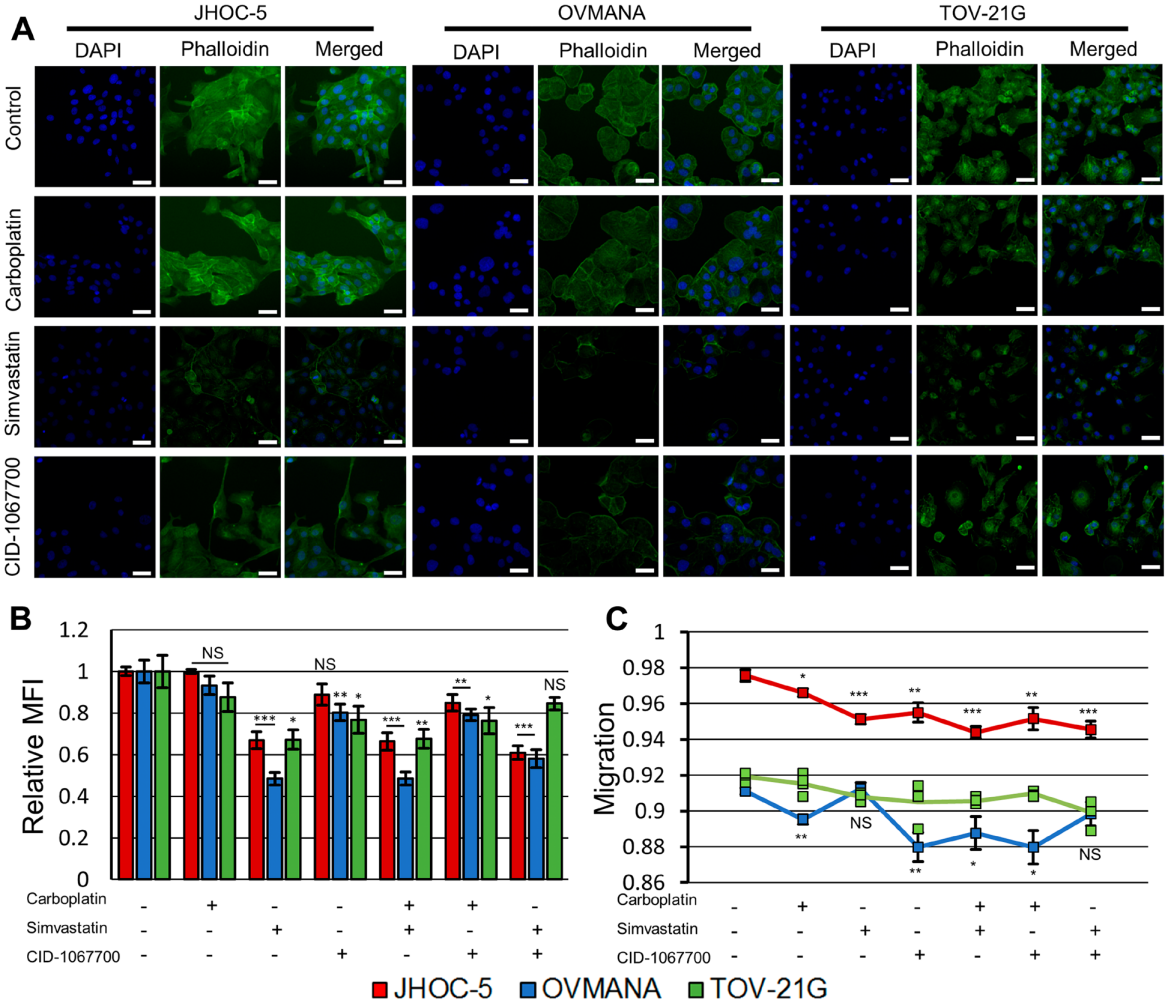
Effect of treatments upon cytoskeletal integrity and migration in JHOC-5, OVMANA and TOV-21G cell lines. (**A**) Representative fluorescence images of JHOC-5, OVMANA and TOV-21G. Individual channels for phalloidin and DAPI were background corrected against the corresponding channel in the respective cell line DMSO control. White scalebar is 50 μm. (**B**) Relative mean fluorescence intensity (MFI) of fluorescently labeled F-actin for JHOC-5 (Red), OVMANA (Blue) and TOV-21G (Green). MFI is shown relative to untreated controls. Results are from two experiments in quadruplicates. Error bars are SEM (*N* = 8). All comparisons are against untreated corresponding cell line controls. (**C**) Migration for JHOC-5 (Red), OVMANA (Yellow) and TOV-21G (Green). Results are from two experiments in quadruplicate (JHOC-5, OVMANA), or one experiment in quadruplicate (TOV-21G) (*N* = 8 for JHOC-5 and OVMANA. *N* = 4 for TOV-21G). Error bars are SEM. All comparisons are against untreated corresponding cell line controls. No comparisons were performed for TOV-21G. ^*^ *p* < 0.05, ^**^ *p* < 0.01, ^***^ *p* < 0.001.

Following treatments with simvastatin or CID-1067700, JHOC-5 cells displayed altered morphology compared to controls treated with DMSO and phalloidin staining revealed a reduction in actin filaments (Figure 2A, Supplementary Figure 1). Simvastatin caused a decrease in mean fluorescent intensity (MFI) staining of actin filaments to a greater extent than CID-1067700 (Figure 2B). This effect was also observed in combinations with carboplatin. The migration of JHOC-5 cells decreased following all treatments, and this decrease was most significant in treatments containing simvastatin (Figure 2C).

OVMANA cells did not display any clear morphologic changes, but reduced actin filament staining similar to the effect observed in JHOC-5 cells upon treatments with simvastatin or CID-1067700 (Figure 2A, Supplementary Figure 1). These effects were observed for both single agents and combination treatments. The reduction in phalloidin staining of actin was similar to JHOC-5 cells but more pronounced in OVMANA cells in response to treatments with simvastatin (Figure 2B). All treatments except treatments with simvastatin as a single agent and the combination of simvastatin and CID-1067700 reduced the migration of OVMANA cells (Figure 2C).

TOV-21G cells displayed morphologic changes and reduced actin staining similar to JHOC-5 cells with treatments with simvastatin or CID-1067700 (Figure 2A, Supplementary Figure 1), however this effect was not observed for the combination of simvastatin and CID-1067700 (Figure 2B). These effects were also observed for combinations with carboplatin, but not for the combination of simvastatin and CID-1067700 (Figure 2B). A non-significant tendency towards decreased migration upon treatment with either drug alone or in combination was observed, similar to the observation in JHOC-5 cells (Figure 2C).

Overall, simvastatin caused disruption of actin filaments in all OCCC cell lines while also significantly reducing the migratory potential in JHOC-5 cells.

### Cell death, cell cycle distribution and other molecular responses

We next investigated the nature of the observed cell death; specifically we aimed to explore whether targeting of Rho GTPases induced caspase-dependent apoptosis and how treatments affected c-Myc levels, which are often increased in OCCC [36, 37].

**Figure 3:**
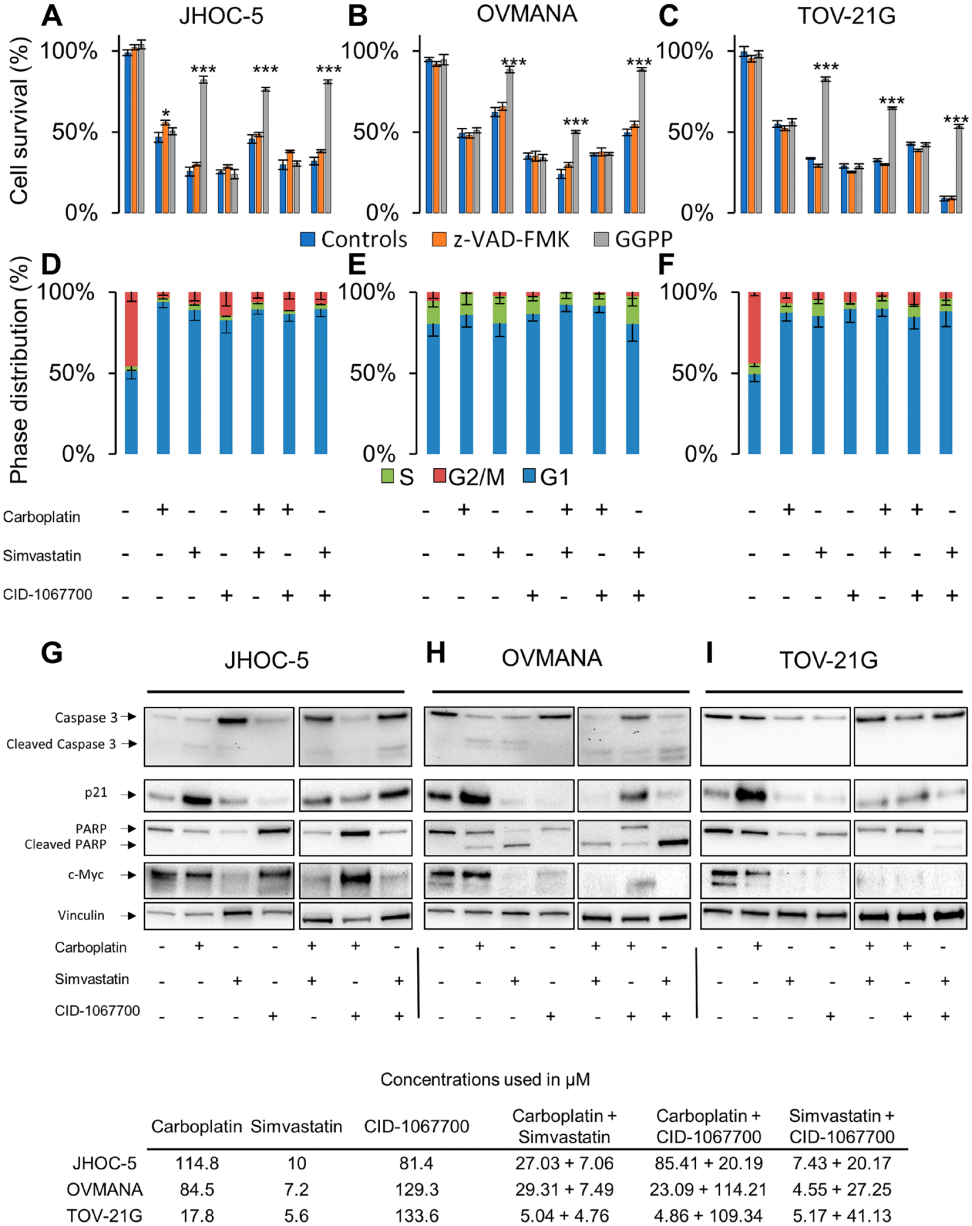
Cellular response to single agent or combination treatments in JHOC-5, OVMANA and TOV-21G cells. Top panel: Cell survival in response to co-treatment with controls (Blue), Z-VAD-FMK (Orange), or GGPP (Grey) in (**A**) JHOC-5: (**B**) OVMANA; (**C**) TOV-21G. Error bars are SEM and results are from at least 3 experiments in triplicate (*N* > 9). Comparisons are made against treated controls. ^*^ *p* < 0.05, ^**^ *p* < 0.01, ^***^ *p* < 0.001. Mid panel: Cell cycle phase distribution in response to treatment for (**D**) JHOC-5; (**E**) OVMANA; (**F**) TOV-21G. Error bars are SD and results are from three independent experiment each with 10,000 cells (*N* = 3). Bottom panel: Immunoblot analysis of single agent treatment in (**G**) JHOC-5; (**H**) OVMANA; (**I**) TOV-21G. Immunoblots were done at least three times for consistency. Concentrations used in the experiments are listed in the table at the bottom.

The addition of the geranylgeranyl precursor GGPP was found to rescue JHOC-5 cells from treatments containing simvastatin, while the irreversible pan-caspase inhibitor Z-VAD-FMK alleviated the cell death caused by carboplatin as a single agent (Figure 3A). All treatments induced a G1 arrest in JHOC-5 cells (Figure 3D). A slight induction of cleaved caspase-3 was observed in response to simvastatin and carboplatin both as single agent and in combination, while an upregulation of caspase-3 in response to treatments with simvastatin was observed (*p*-value: 0.00131) (Figure 3G, Supplementary Figure 2). p21 was upregulated in response to carboplatin (*p*-value: 0.0079), while no significant effects were observed in response to treatments with simvastatin and CID-1067700, although a tendency towards lowered p21 was observed (Figure 3G, Supplementary Figure 2). PARP was downregulated in response to carboplatin and simvastatin and their combination (*p*-value: 0.0000162), while no PARP cleavage was observed in response to either of the treatments. CID-1067700 alone did not significantly alter PARP expression across three individual experiments, however a tendency towards increased PARP expression compared to DMSO controls was observed (*p*-value: 0.0934, Supplementary Figure 2). CID-1067700 in combination with simvastatin caused PARP downregulation (*p*-value: 0.0090). c-Myc was reduced in response to treatments containing simvastatin (*p*-value: 0.0351). Loading was confirmed using total protein assessment by the stain-free method (Supplementary Figure 4), but of note the loading control vinculin showed unequal distribution across treatments.

The addition of GGPP led to increased cell survival of OVMANA cells only after treatments with simvastatin, while Z-VAD-FMK had no effect (Figure 3B). No significant effects on cell cycle phase distributions were observed in OVMANA cells, however the accumulation of cells in G1 regardless of treatment has previously been reported [38] (Figure 3E). Treatments with carboplatin and simvastatin induced cleavage of both caspase-3 (*p*-value: 0.0236) and PARP (*p*-value: 0.00000375) (Figure 3H, Supplementary Figure 2), and this was also observed for their combinations. p21 was upregulated in response to treatment with carboplatin, but reduced in response to treatments with simvastatin and CID-1067700, either alone or in combinations (*p*-value: 0.0000102) (Figure 3H, Supplementary Figure 2). c-Myc was reduced in response to treatments with simvastatin and CID-1067700 (*p*-value: 0.00419) (Figure 3H, Supplementary Figure 2). These effects were also observed in combinations with carboplatin.

The addition of GGPP led to increased cell survival in TOV-21G cells treated with simvastatin, while Z-VAD-FMK had no effect (Figure 3C). A similar pattern of G1 arrest following treatments was observed in TOV-21G cells compared to JHOC5 cells (Figure 3F). No cleaved caspase-3 or PARP was observed in TOV-21G cells, but PARP (*p*-value: 0.03073) and caspase-3 (*p*-value: 0.0221) levels were reduced in response to treatments with simvastatin and CID-1067700 (Figure 3I, Supplementary Figure 2). Also, an increase in p21 following carboplatin treatment was detected, but similar to OVMANA cells a reduction was observed in response to treatments with simvastatin and CID-1067700 (*p*-value: 0.00000689). A reduction in c-Myc, similar to what was observed in OVMANA cells, occurred in response to treatments with simvastatin and CID-1067700 (Figure 3I, Supplementary Figure 2) (*p*-value: 0.00569). These effects were observed with single agent simvastatin and CID-1067700, but also combinations thereof.

Taken together, c-MYC was reduced upon simvastatin treatment in all OCCC cell lines. Caspase-3 and/or PARP-1 cleavage was observed in JHOC-5 and OVMANA cells, indicative of apoptosis.

### Autophagy responses and activation of ERK and AKT

To further evaluate the mechanisms responsible for the cellular responses we investigated the downstream effectors ERK and AKT as well as the autophagy markers p62 and LC3A/B-I/II. These autophagy markers have previously been investigated in HGSOC following statin treatment [39, 40].

**Figure 4:**
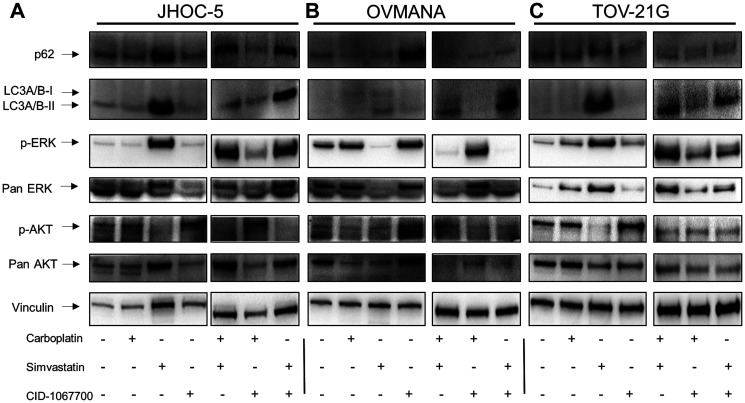
Cellular response to single agent treatments with carboplatin, simvastatin or CID-1067700 or combinations thereof. Immunoblot analysis of single agent treatment in (**A**) JHOC-5; (**B**) OVMANA; (**C**) TOV-21G. Immunoblots were done at least three times for consistency for p-ERK, ERK, p-AKT and AKT. Immunoblots for p62 and LC3A/B-I/II were performed twice for single agent treatments and once for combination treatments. NB a small air bubble partially obscuring an unspecific band in the p-AKT blot in the OVMANA cell line does not affect the interpretation of the result.

Increased levels, although not statistically significant, of both p62 and LC3A/B-II were observed in JHOC-5 cells after simvastatin treatment (Figure 4A, Supplementary Figure 3). p-ERK increased (*p*-value: 0.00166), while a tendency towards decreased p-AKT was observed after treatments with simvastatin either alone or in combinations, however this was not statistically significant (Figure 4A, Supplementary Figure 3).

A tendency towards an increase in p62 was observed in OVMANA cells after treatment with CID-1067700. Additionally, a tendency towards an increase in LC3A/B-I/II following simvastatin treatments was also observed. There was a decrease in both p-ERK (*p*-value: 0.00000606) and total ERK levels after treatments with simvastatin either alone or in combinations (Figure 4C), while no changes were observed for either p-AKT or total AKT.

TOV-21G cells displayed changes in protein levels similar to JHOC-5 cells; LC3A/B-I/II and p-ERK (*p*-value: 0.00863) levels increased. A tendency towards decreased p-AKT (*p*-value: 0.0618) after treatments with simvastatin was observed (Figure 4C, Supplementary Figure 3). p62 levels were not affected in response to any treatments.

The autophagy markers p62 and LC3A/B-I/II showed a tendency towards increased levels to a varying extent in the OCCC cell lines after treatments containing simvastatin. p-ERK levels increased in JHOC-5 and TOV-21G cells and decreased in OVMANA cells upon simvastatin treatment. Effects on p-AKT were not significant.

Interestingly, while equal sample loading on gels was confirmed through stain-free analysis of membranes (Supplementary Figure 4) this was not reflected by the level of expression of the selected proteins evaluated as loading controls. GAPDH, vinculin and tubulin were all affected by the treatments to varying extents (Figure 3G–3I, Figure 4A–4C, Supplementary Figure 4) indicating drug-induced changes in the protein levels of these house-keeping genes and suggesting that effects on the cytoskeleton may render these loading controls less reliable when studying drugs affecting the cytoskeleton.

## DISCUSSION

We recently reported that Rho GTPases and their regulators were differentially expressed in OCCC compared to the other main EOC histotypes [10]. We therefore hypothesized that drugs targeting Rho GTPases and their activity might be beneficial for the treatment of OCCC. In this study we examined the potential of simvastatin treatment in three OCCC cell lines and compared the effects to standard chemotherapy treatment with carboplatin as well as treatment with the Rho GTPase inhibitor CID-1067700. The OCCC cell lines were chosen to reflect the heterogeneity of OCCC, while also reflecting common features of OCCC [41]. A chemo-sensitive HGSOC cell line was also included for reference purposes.

Our data demonstrated that all three OCCC cell lines were significantly more sensitive to simvastatin compared to the HGSOC Caov3 cells. In contrast, the OCCC cell lines JHOC-5 and OVMANA were resistant to carboplatin. The resistance towards carboplatin displayed by JHOC-5 and OVMANA cells may be due to prior exposure to cisplatin treatment in the patients causing acquired resistance or, alternatively, inherent resistance [29, 30]. Despite the differences between the OCCC cell lines our study demonstrates a higher sensitivity towards simvastatin in all OCCC cell lines; further studies will be required to compare between EOC subtypes.

Differences in treatment response between OCCC cell lines may also be explained by differences in ethnicity, however this remains speculative. Both JHOC-5 and OVMANA cells were of Japanese origin, while TOV-21G was Canadian. In this context, OCCC is twice as frequent in Asia compared to the US [42], but the underlying cause between these possible ethnic differences is still not understood and remains to be investigated. From a clinical point of view, only OVMANA cells were positive for both HNF1-β and Napsin A, markers used to diagnose OCCC, while both TOV-21G and OVMANA cells had mutations in the frequently reported genes *ARID1A* and *PIK3CA* [6], underscoring the molecular heterogeneity within this histotype [42].

Oncological treatment paradigms have shifted from single agent treatments to combination treatments including targeted treatments, and we therefore evaluated the effect of pair-wise combinations of the three drugs. Interestingly, we observed antagonistic tendencies when combining either CID-1067700 or simvastatin with carboplatin in all of the cell lines, while a combination of CID-1067700 and simvastatin was generally additive or synergistic, further supporting the concept of Rho GTPase interference in OCCC.

To further understand the underlying cellular mechanisms, we investigated the treatment-induced effects on cytoskeletal integrity and migratory capacity. Both simvastatin and CID-1067700 significantly reduced actin filament staining as demonstrated by the decreased MFI in both single agent and combination treatments and affected the morphological appearance and structure of OCCC cells, while also inhibiting migration, suggesting that both drugs exert their activity by inhibiting Rho GTPases. Adding GGPP to simvastatin rescued all cell lines from cell death, further suggesting that simvastatin interferes with small GTPases [19], but the caspase inhibitor Z-VAD-FMK only rescued carboplatin-induced cell death in JHOC-5 cells, strengthening the notion of differences in the mode of cell death. The effect upon invasive potential was not investigated in this study.

Since amplification of *c-MYC* plays a key role in cancer progression and proliferation and is common in OCCC [36, 37] it is a potential treatment target. As a common denominator, we observed an upregulation of p21 in response to treatment with carboplatin and a reduction of c-Myc after simvastatin treatments, the latter either alone or in combinations, consistent with an arrest in the G1 phase of the cell cycle for JHOC-5 and TOV-21G cells as also reported in HGSOC cell lines [23, 40]. However, we also observed evidence of an activation/disruption of autophagy through the markers p62 and LC3A/B-II, suggesting that simvastatin treatment might work through interfering with autophagy responses in JHOC-5 and TOV-21G cells. This is in line with a report showing impaired autophagosome formation after c-Myc inhibition [43] and is further reinforced by our findings of an increase in p-ERK after simvastatin treatment due to autophagy proteins regulating p-ERK levels [44]. In addition, inhibition of mTOR has been shown to increase autophagy in endometrial cancer, consistent with our findings of reduced p-AKT levels [45]. We also investigated HIF-1α as a marker for induced autophagy; however data for HIF-1α were inconclusive for all cell lines. Taken together, and considering also the disruption of cytoskeletal actin filaments, these data suggest that JHOC-5 and TOV-21G cells may respond to simvastatin treatment through induction of autophagy.

Cleavage of caspase-3 was observed in JHOC-5 cells in response to both carboplatin and simvastatin but not in TOV-21G cells and no cleavage of PARP was observed in either cell line, possibly due to rapid degradation of cleaved products from both PARP and caspase-3. This remains to be investigated.

OVMANA cells did not display evidence of autophagy induction after simvastatin treatment, rather a caspase-mediated apoptotic response. Our data suggest that simvastatin acts through reduction of c-Myc, thereby preventing cell proliferation and possibly inducing cell death. This hypothesis is supported by a report in which c-Myc depletion led to cell cycle arrest in cancer cells at various stages depending on cell type [46]. G1 arrest and subsequent apoptosis is a possible mechanism in OVMANA cells, supported by the presence of cleaved PARP and caspase-3 upon carboplatin and simvastatin treatment. However, while CID-106770 also induced cell death, it was likely due to other mechanisms of action despite a reduction of c-Myc levels due to the absence of cleaved caspase-3 and PARP in both OVMANA and TOV-21G cells.

Interestingly, while vinculin levels reflected loading in OVMANA and TOV-21G cells, simvastatin caused an increase in the levels of loading control proteins in the JHOC-5 cell line, despite equal loading being assured by evaluation of total protein using the stain-free gel. Moreover, neither GAPDH nor tubulin was useful as a loading control, as levels varied in all cell lines after treatment. These coincidental findings may reflect the fundamental association of both tubulin and vinculin with the cytoskeleton, such that caution needs to be taken when selecting loading controls for the study of cytoskeletal integrity.

Whether cytoskeletal disruption and a reduction in c-Myc are directly and completely responsible for the observed cytotoxicity remains to be investigated, but Taté *et al*. (2017) reported that simvastatin cytotoxicity was elicited via cytoskeletal destruction, supporting our findings [47]. Also Robinson *et al.* (2013) reported that statins elicit a dual role as they could both inhibit and initiate autophagy in ovarian cancer, and that these mechanisms likely contribute to the cytotoxic effects of statins [48]. The difference in cellular responses between the cell lines, e.g., in migration, could be associated with differences in both their pheno- and genotypes, as OVMANA and TOV-21G cells harbor co-existing *ARID1A/PIK3CA* mutations, genes reported to be mutated in OCCC, whereas JHOC-5 cells do not. In this context a study by Abou-Taleb *et al*. (2016) reported two different prognostic subtypes of OCCC depending on the protein expression of SWI/SNF complex proteins [49]. However, additional studies would be required to investigate this.

While HGSOC has been studied intensively, OCCC remains a rare subtype with poor prognosis, but our study, although investigative, demonstrates a potential for simvastatin treatment in OCCC. Simvastatin could act through Rho GTPase interference as simvastatin affects the cytoskeletal integrity of OCCC cells at levels which can be achieved in plasma [50]. However, the mechanism is different from Rho GTPase inhibition by CID-1067700. Furthermore, caution should be given, as our data suggest that a combination with standard chemotherapy may elicit an antagonistic response. Whether this is of clinical relevance for patients receiving statin treatment remains unclear and needs to be investigated further, but simvastatin holds promise as a potential drug candidate in OCCC and warrants further investigation in the clinical setting.

## MATERIALS AND METHODS

### Chemicals

TRITON X-100, Trizma Base, Trichloroacetic acid solution (TCA), Sulforhodamine-B Sodium salt, CID-1067700, Geranylgeranyl pyrophosphate ammonium salt (GGPP), Simvastatin, DMSO, MCDB105 and Medium199 were purchased from Sigma Aldrich (Stockholm, Sweden). Carboplatin was purchased from Selleck Chemicals (SMS-gruppen, Rungsted, Denmark). Phalloidin CruzFluor™ 488 Conjugate was purchased from Santa Cruz (AH diagnostics AB, Solna, Sweden). Penicillin/Streptomycin solution (P/S), Fetal Bovine Serum (FBS), DPBS, DMEM: F12, RPMI1640 and Dulbecco’s Modified Eagle’s Medium (DMEM) were purchased from Nordic Biolabs (Täby, Sweden). Paraformaldehyde 16% w/v (PFA) was purchased from VWR (Spånga, Sweden). DAPI was purchased from Thermo Fisher (Göteborg, Sweden). Pan Caspase inhibitor Z-VAD-FMK was purchased from Promega (Nacka, Sweden).

### Cell lines

OVMANA cells were purchased from the Japanese Collection of Research Bioresources (JCRB) Cell Bank. JHOC-5 cells were obtained from the RIKEN National Bio-Resource Project, and TOV-21G and Caov-3 cells were purchased from ATCC (LGC Standards GmbH, Wesel, Germany). JHOC-5 was cultured in DMEM: F12 (1:1), OVMANA in RPMI1640, TOV-21G in Medium199: MCDB105 (1:1) and Caov3 in DMEM, supplemented with 10% FBS (15% for TOV-21G) and 1% penicillin/streptomycin. Cells were kept in 5% CO_2_ at 37°C. All cell line experiments were performed using cell line passages between 5–25. All cell lines were authenticated at Eurofins (Ebersberg, Germany).

### Cell proliferation assay

Cells were plated at 5,000 cells/well in a 96-MicroWell Nunclon plate (VWR) and left for 24 hours after which they were then treated with drugs for 72 hours, with DMSO as the control. Proliferation was evaluated using the sulforhodamine-B assay as described previously [51]. Concentration response curves and IC50 concentrations for single agent regimens and combination treatments were calculated and analyzed using the drc package (Version 3.0-1) [52] in R (Version 3.3.3) [53]. Total concentrations for combination treatments were established using IC50 concentrations for each independent treatment in a 3:1 or 1:1 ratio for each combination possible; e. g. the total concentration used for a 3:1 combination of carboplatin and simvastatin would be: 3 × IC50(Carboplatin) + 1 × IC50(Simvastatin) = total concentration (μM). An IC50 concentration was calculated for each combination from dose response curves, while knowing the initial ratio allowed for a measurement of the individual drug concentration in each combination. Combination index (CI) values for combination treatments were calculated using the Chou-Talalay method [35].

### Cell migration assay

Cell migration assays were performed using ORIS™ cell stoppers (Platypus Technologies, Tebu-bio, Roskilde, Denmark) in CELLSTAR flat-bottomed 96-well plates (VWR) as described previously [54] with the following modifications: Cells were plated at 30,000 cells/well, stained with DAPI in DPBS with 0.1% Triton X-100 (10 mg/ml diluted 1:10,000). Migration was measured using a Platerunner HD (Trophos, Marseille, France). Cell migration was evaluated after 24 hours using the MRI Wound Healing Tool [55] for ImageJ [56] using default settings. To ensure that migration and not proliferation was measured, we compared the number of cell nuclei between controls at baseline and at 24 hours to ensure no significant increase in cell number using the inherent Analyze Particles function in ImageJ with default settings.

### Evaluation of cytoskeletal integrity

Cells were plated in CELLSTAR flat-bottomed 96-well plates at 5,000 cells/well and allowed to attach for 24 hours, then treated with drugs for 72 hours, after which the cells were fixed with 4% PFA. Cells were stained with DAPI (10 mg/ml diluted 1:10,000) and Phalloidin CruzFluor™ 488 Conjugate (1:1000) in DPBS with 0.1% Triton X-100. Fluorescent intensities were measured using a Cellomics ArrayScan VTI HCS reader from Thermo Scientific (Weltham, MA, USA). The mean fluorescence intensity (MFI) of phalloidin was measured across the nucleus as identified by the DAPI stain and an average was calculated of at least 500 cells/well unless otherwise stated.

### Cell cycle analysis

Cells were plated in 6-well plates (VWR) at 200,000 cells/well and allowed to attach for 24 hours, and subsequently treated with drugs for 72 hours. Cells were prepared as previously described [57] with the following modifications: Cells were resuspended in 0.5 ml DPBS containing 50 μg/ml propidium iodide (PI) (Sigma Aldrich), 100 μg/ml RNase A (Qiagen, Sollentuna, Sweden) and 0.1% Triton X-100. Cell cycle distribution was analyzed using a FACSverse (BD Biosciences, Stockholm, Sweden) and a total of 10,000 cells were analyzed for each sample. Post-analysis was performed using the BD FACSuite™ software (Version 1.0.6) (BD Biosciences).

### Immunoblot analysis

Cells were plated in 100 mm dishes (VWR) at 750,000 cells/plate and allowed to attach for 24 hours, and subsequently treated for 72 hours, after which cells were collected by scraping and lyzed using RIPA buffer (Sigma Aldrich) containing Halt™ Protease Inhibitor (Thermo Fisher). Twenty-five μg of proteins (assessed by Pierce™ BCA Protein Assay (Thermo Fisher)) were separated using the Bio-Rad stain-free protocol [58] with the following modifications: PVDF membranes were cut to evaluate multiple proteins in parallel and blocked in either 5% Blotting-Grade Blocker in TBS-T 0.05% (Bio-Rad) or 5% BSA in TBS-T 0.05% (Sigma Aldrich). Chemiluminescent signal was captured using Clarity Western ECL Substrate (Bio-Rad) and the ChemiDoc XRS+ system (Bio-Rad). For reprobing, blots were stripped using Restore™ PLUS Western Blot Stripping Buffer (Thermo Fisher). Raw TIFF files were analyzed and pixel densities were quantified using ImageJ [56]. The following polyclonal antibodies were used: Caspase-3 (#9662, 1:500), PARP (#9542, 1:1,000), p21 (#2947, monoclonal, 1:1,000), p-AKT (#9271, 1:1,000), p-ERK (#4370, 1:1,000), AKT (#9272, 1:1,000), LC3A/B I/II (#4108, 1:500), GAPDH (#5174, 1:10,000) and α/β-Tubulin (#2148, 1:1,000) (Cell Signaling Technology, BioNordika, Stockholm, Sweden). ERK (sc-292838) (Santa Cruz, 1:1,000). P62 (GP62-C-WBC, 1:1,000) (Progen, Heidelberg, Germany). Vinculin (9131, 1:10,000) (Sigma Aldrich). Monoclonal c-Myc (ab32072, 1:1,000) (Abcam, Cambridge, UK). Secondary polyclonal antibodies were mouse (#31430, 1:10,000) or rabbit (#31460) (Thermo Fisher, 1:10,000).

### Cell line immunocytochemistry (ICC)

Two million cells were formalin-fixed and paraffin-embedded. ICC was performed for Napsin A (1:20) (Leica, Trio-lab, Mölndal, Sweden) and HNF1-β (Sigma Aldrich) using the standard IHC protocol at the Department of Clinical Pathology, Division of Laboratory Medicine, Skåne University Hospital, Lund, Sweden. Evaluation was performed by a trained gynecological pathologist.

### Statistics

All experiments were performed in triplicates and repeated at least three times unless otherwise noted. All statistical testing was done using R version 3.3.3 [53] and the FSA package version 0.8.25 [59]. A one-way ANOVA test was used to test for significant differences between samples. Multiple testing was accounted for using the Dunnett’s test with a significance threshold of 0.05. IC50 concentrations were compared using the compParm ()-function (*Z*-test) in the drc-package in R [52], and multiple testing was adjusted for using Bonferroni correction. All error bars are standard error of the mean (SEM) unless otherwise noted. Significance thresholds are depicted as follows ^*^ *p* < 0.05, ^**^ *p* < 0.01, ^***^ *p* < 0.001.

## SUPPLEMENTARY MATERIALS



## Abbreviations

AKT: Protein Kinase B Alpha
ARID1A: AT-Rich Interaction Domain 1A
c-Myc: MYC Proto-Oncogene, BHLH Transcription Factor
EOC: Epithelial ovarian cancer
ERK: Mitogen-Activated Protein Kinase 1
GGPP: Geranylgeranyl pyrophosphate
HGSOC: High-grade serous ovarian cancer
HNF1-β: HNF1 Homeobox B
IC50: Inhibitory concentration at 50% effect
KRAS: KRAS Proto-Oncogene, GTPas
LC3A/B-I/II: Microtubule-associated proteins 1A/1B light chain 3A (I/II)
NAPSIN A: Napsin A Aspartic Peptidase
OCCC: Ovarian clear cell carcinoma
p21: Cyclin-dependent kinase inhibitor 1
p62: Nucleoporin 62
PARP: Poly(ADP-Ribose) Polymerase 1
PI: Propidium iodide
PIK3CA: Phosphatidylinositol-4,5-Bisphosphate 3-Kinase Catalytic Subunit Alpha
PTEN: Phosphatase and Tensin Homolog
