# Supplementary material for: Simvastatin is a potential candidate drug in ovarian clear cell carcinomas
Source: Oncotarget. 2020 Oct 6;11(40):3660–74. doi: 10.18632/oncotarget.27747 (PMC7546754; doi:10.18632/oncotarget.27747)
Supplement: Supplementary file 1 [file oncotarget-11-3660-s001.pdf]

Simvastatin is a potential candidate drug in ovarian clear cell carcinomas

SUPPLEMENTARY MATERIALS

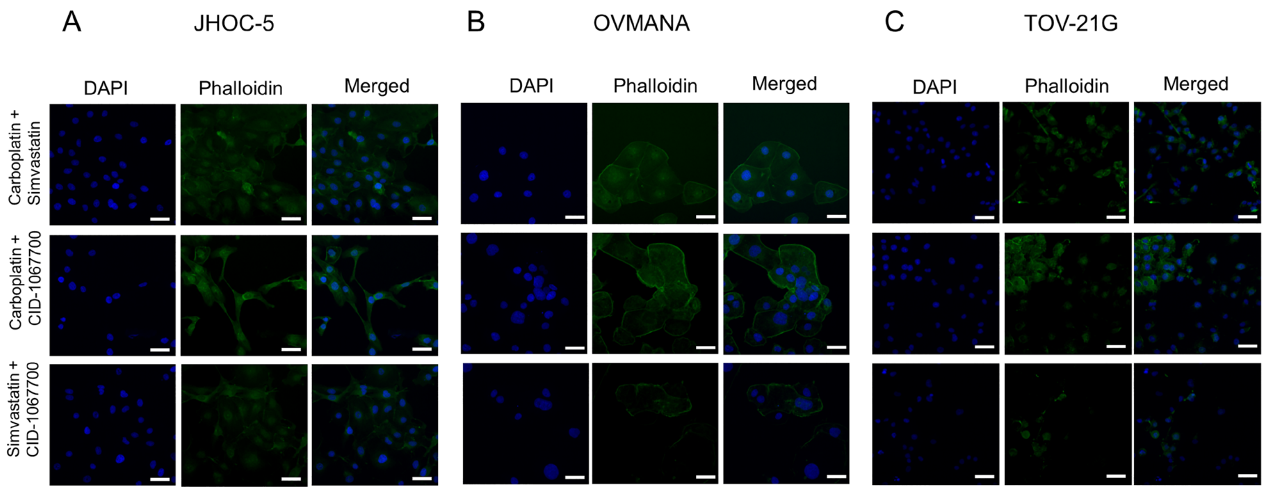

Supplementary Figure 1: Effects of combination treatments upon the cytoskeleton. Fluorescence images of (**A**) JHOC-5, (**B**) OVMANA and (**C**) TOV21-G. Individual channels for phalloidin and DAPI were background corrected against the corresponding channel in the respective cell line DMSO control (Figure 2). White scale bar is 50 µm.

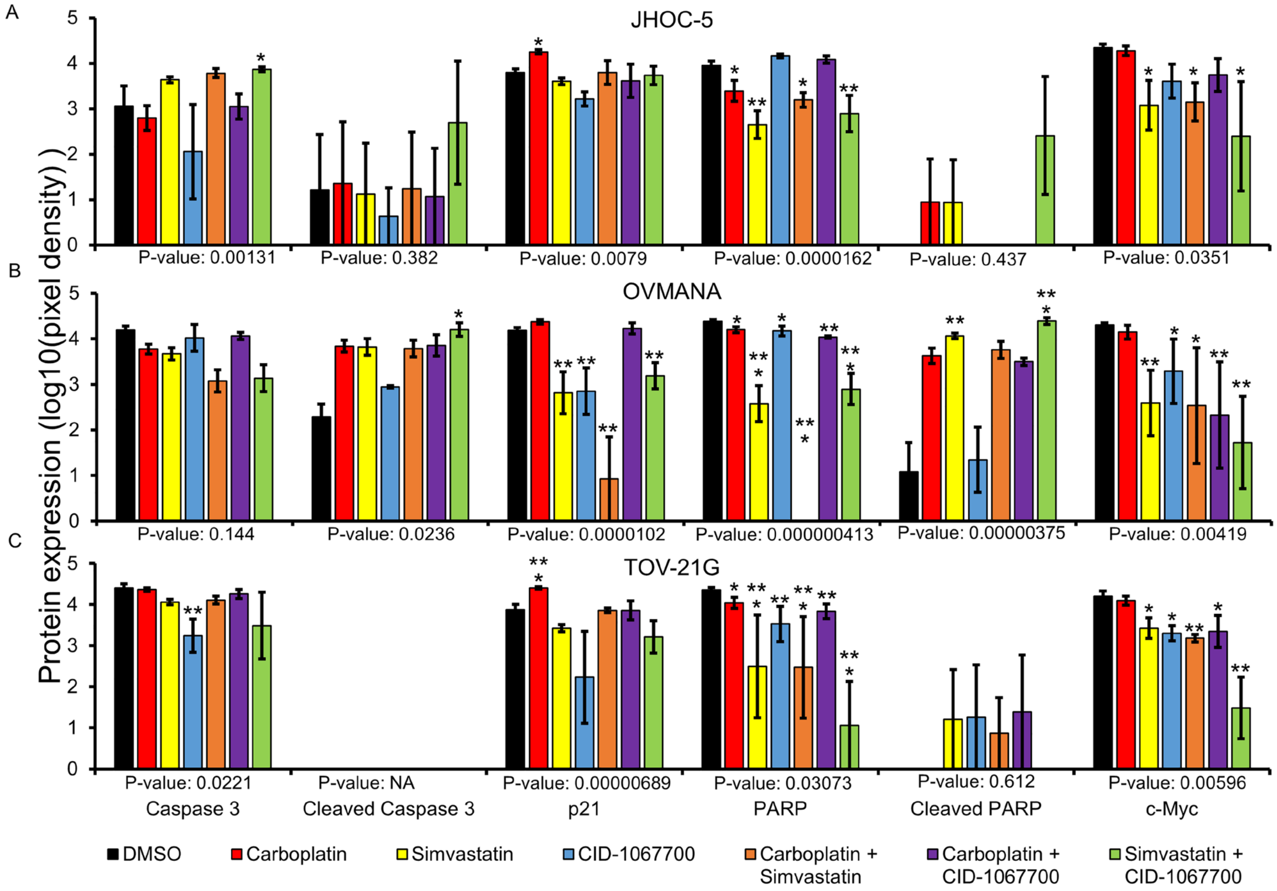

Supplementary Figure 2: Bar plot of protein expression levels for Figure 3G–3I. (**A**) JHOC-5, (**B**) OVMANA and (**C**) TOV21-G, for Caspase 3, Cleaved Caspase 3, p21, PARP, Cleaved PARP and c-Myc for the following treatments: DMSO (Black), Carboplatin (Red), Simvastatin (Yellow), CID-1067700 (Blue), Carboplatin+Simvastatin (Orange), Carboplatin+CID-1067700 (Purple) and Simvastatin+CID1067700 (Green). Error bars are standard error of the mean. Dunnett's Test was used to correct for multiple testing with each treatment being compared to the corresponding DMSO control. *P*-values listed below each bar plot are the corresponding One-way ANOVA test (six degrees of freedom). $^{*}p < 0.05$; $^{**}p < 0.01$; $^{***}p < 0.001$.

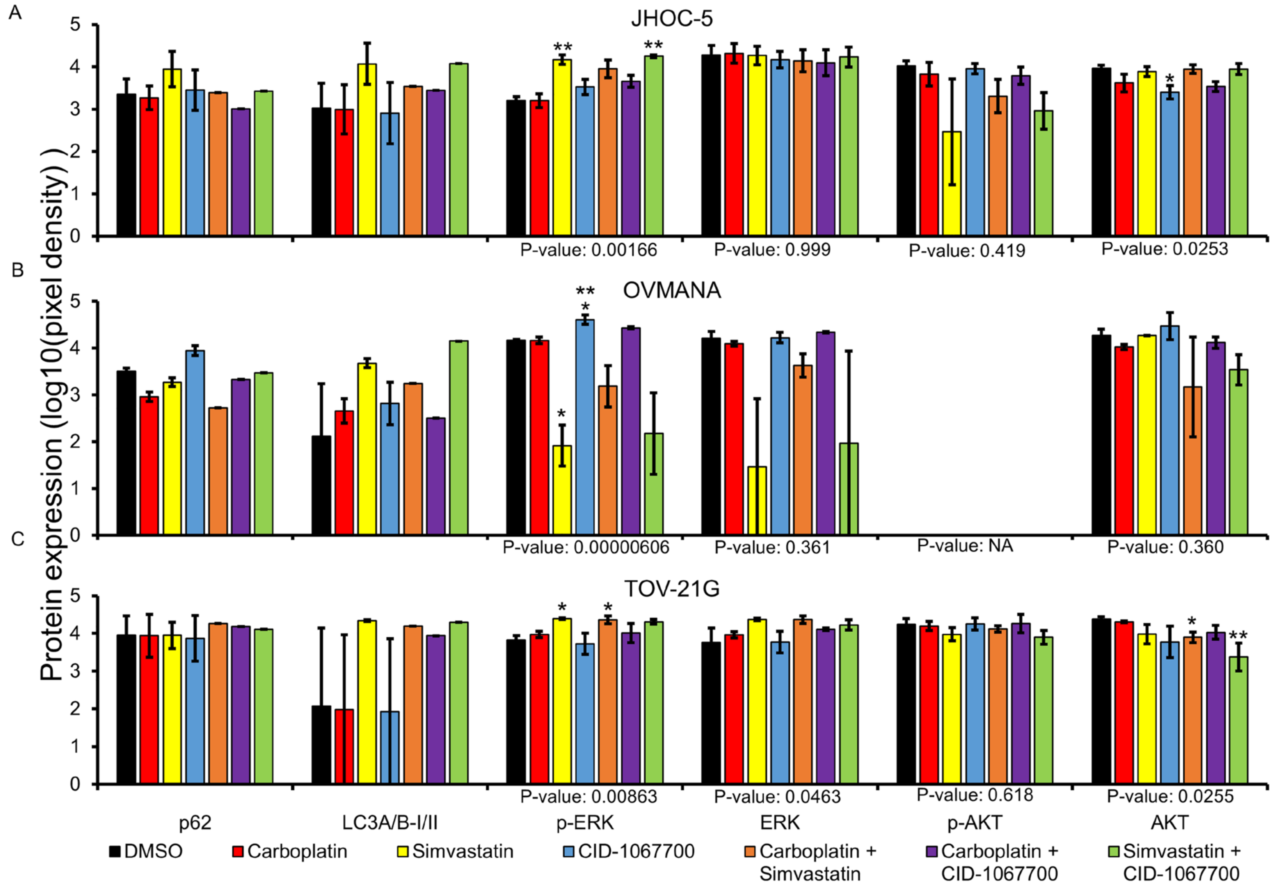

Supplementary Figure 3: Bar plot of protein expression levels for Figure 4A–4C. (**A**) JHOC-5, (**B**) OVMANA and (**C**) TOV21-G, for p62, LC3A/B-I/II, p-ERK, ERK, p-AKT and AKT for the following treatments: DMSO (Black), Carboplatin (Red), Simvastatin (Yellow), CID-1067700 (Blue), Carboplatin+Simvastatin (Orange), Carboplatin+CID-1067700 (Purple) and Simvastatin+CID1067700 (Green). Error bars are standard error of the mean. Dunnett's Test was used to correct for multiple testing with each treatment being compared to the corresponding DMSO control. *P*-values listed below each bar plot are the corresponding One-way ANOVA test (six degrees of freedom). No significance testing was performed for p62 and LC3A/B-I/II. ANOVA: $^{*}p < 0.05$; $^{**}p < 0.01$; $^{***}p < 0.001$.

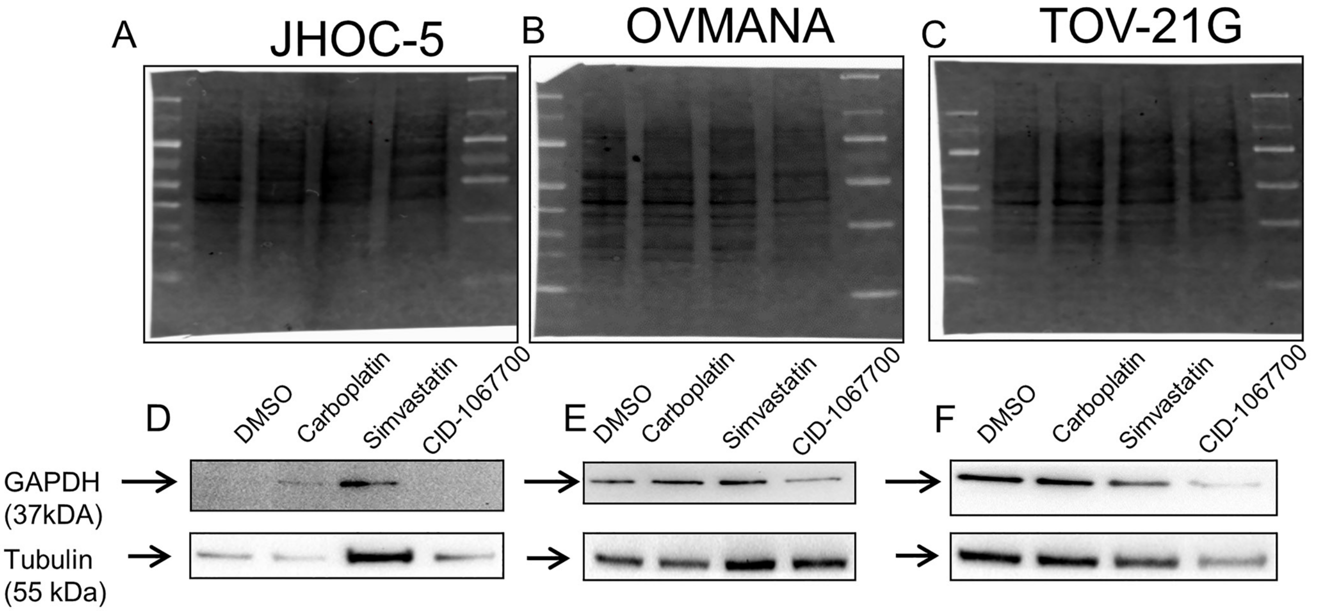

Supplementary Figure 4: Immunoblot loading controls for single drug treatments. Top panel: Stain-free membrane loading controls of total protein for: (**A**) JHOC-5, (**B**) OVMANA and (**C**) TOV21-G. Bottom panel: Immunoblots of GAPDH and Tubulin for (**D**) JHOC-5, (**E**) OVMANA and (**F**) TOV21-G. Results are from at least two experiments.
